# The Completed PacBio Single-Molecule Real-Time Sequence of *Methylosinus trichosporium* Strain OB3b Reveals the Presence of a Third Large Plasmid

**DOI:** 10.1128/genomeA.01349-17

**Published:** 2017-12-07

**Authors:** John R. Heil, Michael D. J. Lynch, Jiujun Cheng, Ola Matysiakiewicz, Maya D’Alessio, Trevor C. Charles

**Affiliations:** a Department of Biology, University of Waterloo, Waterloo, Ontario, Canada; b Metagenom Bio Inc., Toronto, Ontario, Canada

## Abstract

Presented here is the complete genome sequence of the well-studied *Rhizobiales* methanotroph *Methylosinus trichosporium* strain OB3b. The assembly contains 5,183,433 bp, corresponding to a chromosome of 4,508,832 bp and three circular plasmids of 285,280 bp, 209,102 bp, and 180,219 bp.

## GENOME ANNOUNCEMENT

*Methylosinus trichosporium* strain OB3b (1), a member of the *Methylocystaceae* family within the *Rhizobiales* order of *Alphaproteobacteria*, is an obligately aerobic methanotroph that has served as a model for the study of methanotrophy and the related ability to degrade hydrocarbons (2). A draft genome sequence, reported in 2010 (3), was the first one of a member of the *Methylocystaceae* family. This sequence included a single circular chromosome and two large plasmids. Transcriptomic and metabolomic studies have been facilitated by this draft sequence (4, 5).

In our quest to develop genome engineering methods for strain OB3b, we recognized the limitations brought on by lack of a completed genome sequence. We therefore decided to have the genome completed using PacBio single-molecule real-time (SMRT) long-read technology. A culture of strain OB3b was a gift of Jeremy Semrau. Colonies were grown on nitrate mineral salts (NMS) agar incubated at 30°C in a methane-air atmosphere. DNA was isolated from pooled colonies by using a cetyltrimethylammonium bromide (CTAB)-phenol-chloroform extraction method (6). The method was modified slightly; the SDS concentration during lysis was increased to 1%, and a chloroform extraction step was added after the phenol-chloroform extraction. The DNA was provided to McGill University and the Génome Québec Innovation Centre, where a sheared large-insert library was prepared and sequenced on the Pacific Biosciences RS-II SMRT instrument. The subread count was 132,509, with a mean length of 7,199, for a total of 953,876,416 bases, giving approximately 180-fold coverage. The raw-read quality score was 85.

Contigs were assembled by the sequencing facility using the Hierarchical Genome Assembly Process (HGAP) workflow. Briefly, raw subreads were extracted from the bax.h5 data files, and a preassembly was generated using BLASR (7). This preassembly then seeded the Celera assembler (8). Raw reads were aligned to the resulting contigs using BLASR, and high-quality consensus sequences were generated through Quiver variant calling. The assembly was circularized by the Circlator program (9) using the raw reads corrected by Canu v.1.6 (10). One unitig (corresponding to GenBank accession number CP023739) was not initially circularized despite an apparent 15-kb repeat at each end. A manual “break” was introduced roughly at the midpoint, and the sequences were reassembled using the toAmos and minimus2 (11) programs. The resulting unitig was returned to the original assembly, and Circlator was rerun, resulting in four circular contigs, with a complete genome size of 5,183,433 bp. One contig of 4,508,832 bp corresponded to the chromosomal sequence in the previously determined draft genome but comprises four segments that are joined in an order and orientation that are different than those of the draft sequence. Two of the smaller contigs, 285,280 bp and 180,219 bp, corresponded to the two previously reported plasmids. The remaining contig appears to be a third large plasmid, of 209,102 bp, that was not reported in the draft sequence. Interestingly, sequence reads mapping to this plasmid were not found in the draft sequence or the reads contributing to the draft sequence. The culture whose DNA contributed to the draft sequence was likely lacking this plasmid. Each of the three plasmids contains an iconic *repABC* operon (12).

### Accession number(s).

The genome sequence reported here has been deposited in GenBank under the accession numbers CP023737, CP023738, CP023739, and CP023740 and is a component of BioProject number PRJNA413061.
